# Ultrasensitive detection of telomerase activity in a single cell using stem-loop primer-mediated exponential amplification (SPEA) with near zero nonspecific signal†
†Electronic supplementary information (ESI) available. See DOI: 10.1039/c6sc00802j


**DOI:** 10.1039/c6sc00802j

**Published:** 2016-04-28

**Authors:** Honghong Wang, Hui Wang, Chenghui Liu, Xinrui Duan, Zhengping Li

**Affiliations:** a Key Laboratory of Applied Surface and Colloid Chemistry , Ministry of Education , Key Laboratory of Analytical Chemistry for Life Science of Shaanxi Province , School of Chemistry and Chemical Engineering , Shaanxi Normal University , Xi'an 710062 , Shaanxi Province , P. R. China . Email: liuch@snnu.edu.cn ; Email: lzpbd@snnu.edu.cn

## Abstract

Telomerase is a crucial biomarker for cancers. Its reliable and sensitive detection, particularly in a single cell, has great significance for the early diagnosis of cancers, studies on tumor progression and anticancer therapy, which remain a challenge, due to nonspecific amplification. Herein, we developed a novel stem-loop primer-mediated exponential amplification (SPEA) strategy, which can specifically and efficiently amplify the telomerase-elongated telomere repeat unit with near zero nonspecific signal. The SPEA-based assay can accurately detect telomerase activity in the crude lysate of a single cell and is suited for detecting the cellular heterogeneity arising from cell-to-cell variations.

## Abstract 2

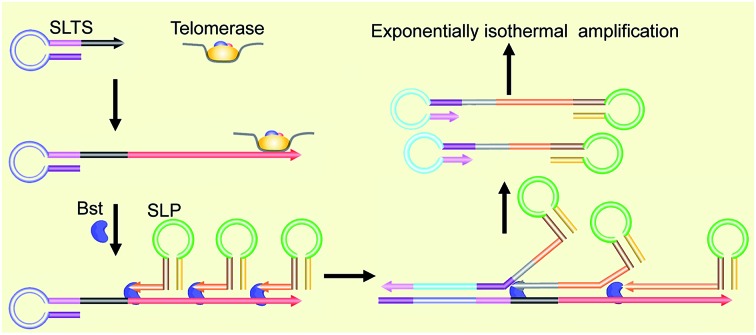
A SPEA strategy is developed for the detection of telomerase activity in a single cell with a near zero nonspecific signal.

## Introduction

In normal somatic cells, chromosomes progressively lose their telomeric sequences, tandem GT-rich repeats (TTAGGG)_*n*_, per cell division due to the DNA end-replication problem.^1^ Eventually, the telomere shortening leads to cellular senescence.^2^ Telomerase is a unique ribonucleoprotein with reverse transcriptase activity, which can add repeated segments of DNA (TTAGGG)_*n*_ to the ends of chromosomes. Telomerase is repressed in most normal somatic cells but is reactivated in the majority of tumor cells,^3^ which allows telomere length maintenance and cell immortalization, and has been considered to be involved in carcinogenesis.^4^ Telomerase is therefore an effective tumor marker and therapeutic target for anticancer treatment. Telomerase activity assay, particularly in a single cell, is of great significance for the early diagnosis of human cancers, studies of tumor progression, and screening anticancer therapy. However, even in tumor cells, telomerase levels are still very low so that the telomerase activity assay needs DNA amplification or signal amplification to achieve satisfactory sensitivity. The PCR-based telomeric repeat amplification protocol (TRAP)^5^ is the most widely applied to detect telomerase activity. Moreover, many modified TRAP assays have been developed to improve quantification and simplify the post-PCR steps.2b,^6^ Unfortunately, the TRAP assays have some apparent weaknesses, such as the requirements of thermal cycles and long amplification time, false positive or negative signals resulting from PCR-derived artifacts, and interferences from proteins present in cell extracts.^7^ To overcome the weaknesses, Plaxco and coworkers have developed the modified TRAP assay using primer-modified gold nanoparticles.^7^ Wright and coworkers have detected telomerase activity by using droplet digital PCR.^8^ These modifications have made great advances for PCR selectivity and sensitivity in complex samples.

Recently, a number of PCR-free assays have also been developed for direct detection of telomerase activity, such as colorimetric and fluorescence detection,^9^ chemiluminescence and electrochemiluminescence methods,^10^ electrochemical detection,^11^ surface plasmon resonance (SPR),^12^ and immunoassay-based methods.^13^ Owing to the lack of an effective amplification mechanism, most of these assays do not achieve sensitivity comparable to the TRAP assay.^14^ Mirkin and coworkers have devised a bio-barcode assay based on telomerase substrate oligonucleotide-functionalized gold nanoparticles,^14^ which has obtained a PCR-like sensitivity with a detection limit of 10 Hela cells. However, the bio-barcode assay needs multiple steps for signal amplification and is a time-consuming detection protocol. More recently, Weizmann,15a,b Zhang,15*c* and their coworkers developed ultrasensitive methods for the detection of telomerase at the single cell level, based on the exponential isothermal amplification (EXIA) of telomerase-elongated telomeric repeat DNA. These methods eliminate the requirement of the thermal cycling procedure and greatly improve the complicated and time-consuming protocols. Nevertheless, the EXIA needs the synergistic reactions catalyzed by DNA polymerase and nicking endonucleases, which increase the expense and inevitably produce nonspecific amplification signals.^15^,^16^

In recent years, single-cell analysis to detect protein biomarkers and gene expression in individual cells has attracted much attention.^17^ The individual cells from the same tissue may actually differ from each other and have different roles that are characterized by significant cellular heterogeneity. Therefore, the detection of cell-to-cell variations in telomerase activity is extremely important to provide deep insight regarding cancer processes, as well as the drug response of tumor cells. Although a few of the telomerase assays mentioned above have reported the detection of telomerase activity at the single cell level, all samples in these assays are extracted from a large number (10^3^ to 10^6^) of cells and are then diluted to be equivalent to a single cell level. Such assays can only detect averaged values of telomerase activity from cell populations, which mask the important information between individual cells.

As described above, exponential DNA amplification (such as EXIA and PCR) is a powerful tool for achieving high sensitivity in telomerase detection. Nonspecific amplification is the key problem limiting the sensitivity and specificity of the assays in detecting telomerase activity.^15^ Herein, by ingeniously designing a stem-loop telomerase substrate (SLTS) and a stem-loop primer (SLP) specific to the telomerase repeat DNA, we can amplify the telomerase-elongated telomere repeat DNA using the stem-loop primer-mediated exponential amplification (SPEA) with near zero nonspecific signal. The SPEA reaction can be performed with only one-type of DNA polymerase under isothermal conditions. Most importantly, the proposed assay can greatly improve the sensitivity and specificity of the detection of telomerase activity, due to the extremely low background and thus, can directly detect telomerase activity from a single cell lysate.

## Results and discussion

### 1. Principle of the SPEA strategy for the detection of telomerase activity

**Fig. 1:**
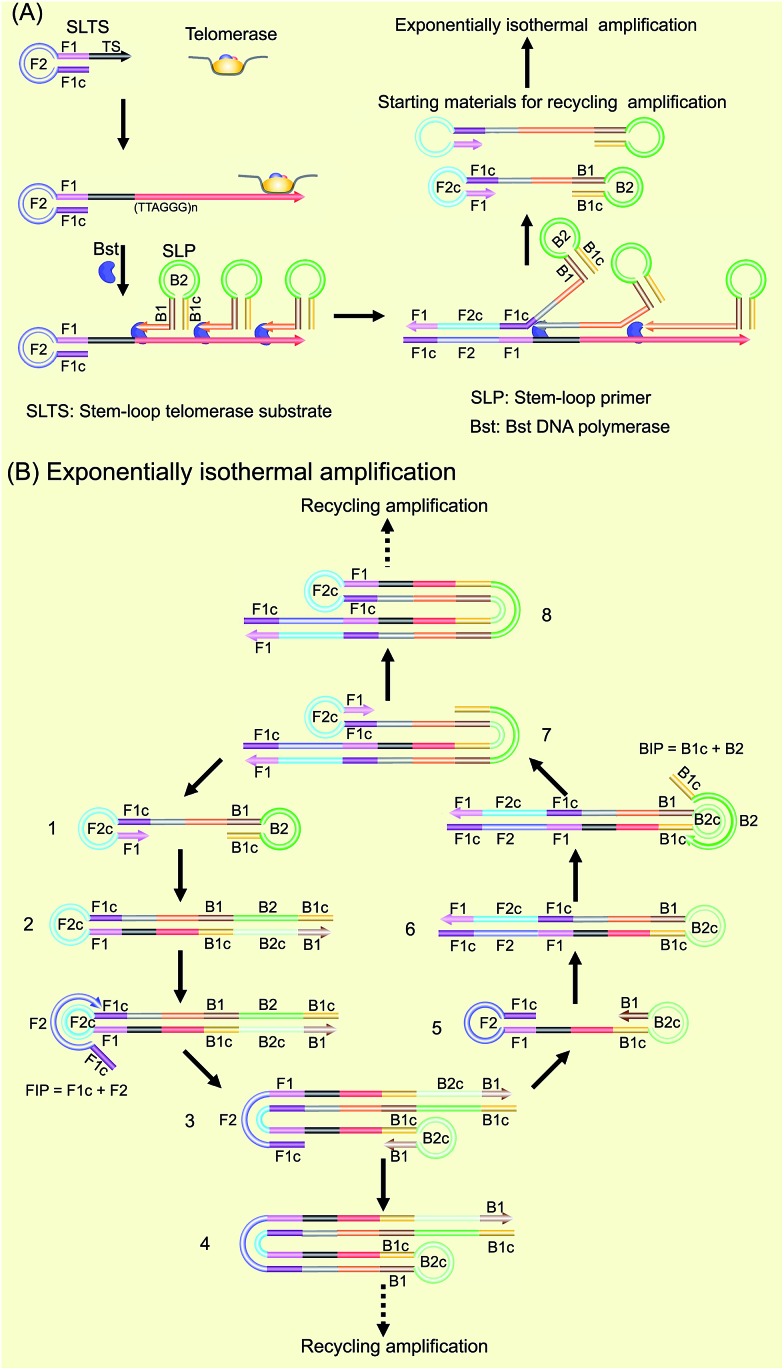
Schematic of the SPEA-based telomerase assay. (A) Illustration of the formation mechanism of the starting materials with the double stem-loop structure for SPEA. (B) Illustration of the recycling mechanism of the SPEA reaction.

Our new strategy for the detection of telomerase activity is schematically illustrated in Fig. 1. Firstly, the SLTS is rationally designed, consisting of a stem-loop structure and the telomerase substrate (TS, AATCCGCGAGCAGAGTT) at its 3′-end. In the presence of telomerase, the TS is elongated with catalysis of the telomerase by adding tandem repeats (5′-TTAGGG-3′)_*n*_ to the 3′-end (arrow showing the direction). Subsequently, the SLP is elegantly designed, consisting of another stem-loop structure and a single strand (ss) DNA at its 3′-end, with a specific sequence (5′-CCCTAACCCTAACCCTAACCC-3′), complementary to more than three repeats. Therefore, the ssDNAs at the 3′-end of the SLPs can hybridize with the repeat DNA side by side and reversely extend along the repeat DNA with the catalysis of Bst DNA polymerase. Owing to the high strand displacement activity of Bst DNA polymerase, the extension of the first SLP can open the stem in the SLTS to form a double strand (ds) DNA. The extension of the ssDNA in the second SLP will displace and release the extension product of the first SLP. Then, extension of the ssDNA in the third SLP is followed up to displace the extension product of the second SLP, and so on. Many SLP-linked ssDNAs with different lengths can be released and new stem-loop structures can be formed at the 3′-ends of these ssDNAs. Therefore, the strand displacement finally produces a series of double stem-loop DNAs with different length ssDNA in between.

The as-produced double stem-loop DNAs are just the starting materials for the amplification stages of loop-mediated isothermal amplification (LAMP), which is well known as an outstanding isothermal DNA amplification method with high sensitivity and specificity.^18^ Therefore, the subsequent amplification steps are the most similar to the amplification stages of LAMP. Once the double stem-loop DNAs are formed, they can initiate the rapid auto-cycling strand displacement DNA synthesis at a constant temperature in the presence of the forward inner primer (FIP) and the backward inner primer (BIP). Firstly, the double stem-loop DNA (material 1) will auto-start the self-primed extension at its 3′-end to form material 2, consisting of a dsDNA and a ssDNA loop (F2c). The FIP is made up of F2 and F1c (c means complementary sequence), which can subsequently hybridize with F2c in material 2 and extend along the upper strand to form a dsDNA and to displace the lower strand to form material 3. The displaced lower strand can quickly be converted to a stem-loop DNA at its 3′-end, which can also perform the self-primed extension to form stem-loop DNA with a longer stem (material 4) and release material 5 with double stem-loop structure. The material 5 then produces material 6 by the self-primed extension, in which the ssDNA loop (B2c) is complementary to B2 in the BIP. Therefore, BIP will hybridize with the ssDNA loop and extend to produce material 7. The self-primed extension of material 7 will further form another stem-loop DNA with a longer stem (material 8) and release material 1 with double stem-loops, which can continuously start the recycling amplification as described above. In addition, the ssDNA loops (B2c and F2c) of material 4 and material 8 can respectively hybridize with BIP and FIP. Through a similar process as demonstrated above, material 4 and material 8 can produce the stem-loop DNAs with longer stems and double stem-loop DNAs with longer ssDNA in between. The double stem-loop DNAs will sequentially form the recycling amplification. The stem-loop DNAs will continuously produce the stem-loop DNAs with longer stems, and the double stem-loop DNAs with longer ssDNA in between, and so on. Therefore, more and more recycling amplification can be formed, which will mediate faster and faster exponential DNA amplification and finally produce a large amount of the mixture of stem-loop DNAs with various stem lengths. Therefore, the LAMP products can be label-free and real-time detected by using SYBR Green I, an effective and inexpensive fluorescence dye for selectively staining dsDNA.

### 2. Analytical performance of the SPEA reaction

**Fig. 2:**
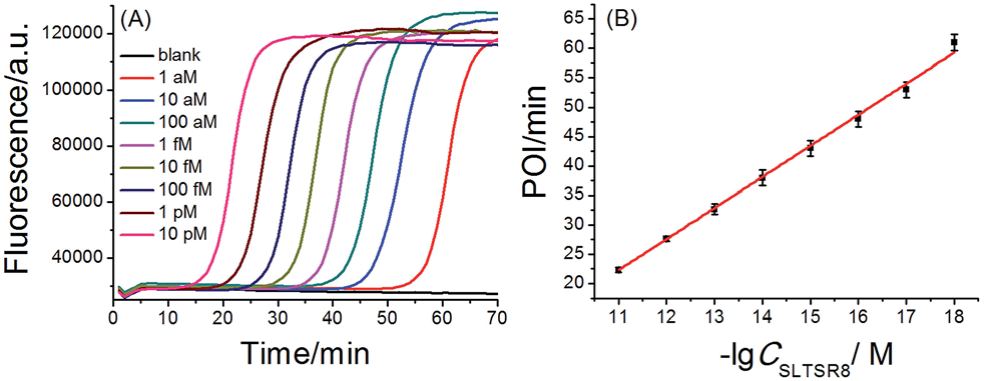
(A) Real-time fluorescence signals of SPEA reactions produced by SLTSR8 with different concentrations. From left to right, SLTSR8 concentration was successively 10 pM, 1 pM, 100 fM, 10 fM, 1 fM, 100 aM, 10 aM, 1 aM and blank. The blank was detected with the same procedures, but without SLTSR8. (B) The linear relationship between the POI values of the corresponding amplification curves and logarithm of the SLTSR8 concentrations. Error bars indicate the standard deviation of three replicative tests.

A synthetic telomerase-elongated product SLTSR8, corresponding to SLTS elongated with eight telomeric repeats (TTAGGG), was firstly employed as a model to optimize the experimental conditions for telomerase detection and to evaluate the performance of the proposed telomerase assay. 100 nM SLP, 0.4 U μL^–1^ Bst DNA polymerase and reaction temperature at 65 °C were found to be optimum for the proposed assay (see Fig. S1–S3 in ESI†). Under the optimum conditions, as shown in Fig. 2A, as low as 1 aM SLTSR8, corresponding to about 6 copies in a reaction volume of 10 μL, can be accurately detected. Well-defined real-time fluorescence signals can also be observed for SLTSR8 in the concentration range of 1 aM to 10 pM with measurement of fluorescence intensity produced by the exponential amplification reaction under isothermal conditions within one hour. The point of inflection (POI), defined as the time corresponding to the maximum slope in the real-time fluorescence curve, is adopted to quantitatively evaluate the performance of the proposed assay. When the POI values are plotted against the logarithm (lg) of SLTSR8 concentrations, as depicted in Fig. 2B, there is an excellent linear relationship in the range of 1 aM to 10 pM. The correlation equation is POI = –35.8 – 5.28 lg *C*_SLTSR8_ (M) with the corresponding correlation coefficient *R*^2^ = 0.998, indicating that the proposed assay has a wide dynamic range, at least over 7 orders of magnitude. It is worth noting that the blank fluorescence signal shown in Fig. 2A is a straight line and remains almost constant. Notably, even when the reaction time was extended up to 240 min, the blank fluorescence maintained a constant straight line (Fig. S4 in ESI†), indicating the near zero nonspecific amplification in the proposed SPEA strategy. The extremely low nonspecific amplification is mainly due to the ingenious probe design and isothermal nature of the exponential amplification in the SPEA system, which has been discussed in detail in the ESI.† The LAMP-based assay has been reported to detect a few copies of nucleic acid molecules.^18^ With the rational design of the proposed SPEA assay, one of the telomerase-elongated SLTS can produce multiple double stem-loop DNAs, the starting materials for LAMP amplification. Therefore, the proposed assay should be theoretically capable of detecting one copy of the telomerase-elongated SLTS. However, when the elongated SLTS is less than 1 aM (*i.e.* less than 6 copies), it is difficult to equably dilute such a small amount of the elongated SLTS in a solution. Therefore, it is difficult to obtain quantitatively accurate results for the detection of target molecules with concentration less than 1 aM in a solution.

### 3. Detection of telomerase activity in Hela cancer cells

**Fig. 3:**
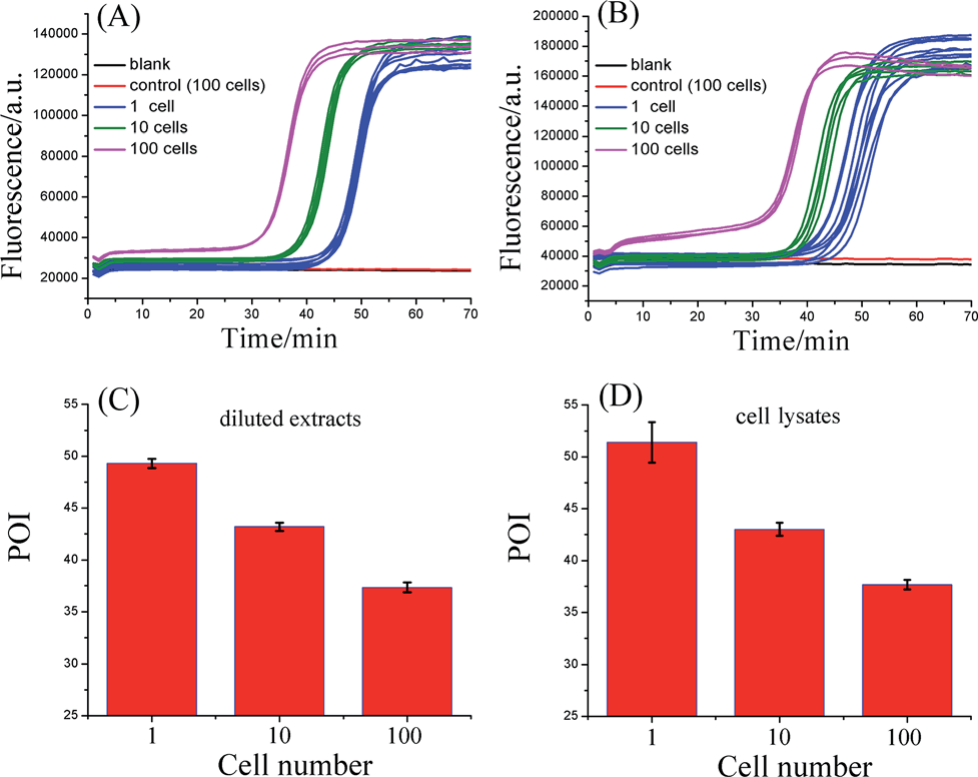
Detection of telomerase activity from the Hela cancer cells. (A) Real-time fluorescence curves for detecting diluted extracts equivalent to 1, 10, and 100 Hela cells. (B) Real-time fluorescence curves for detecting the lysates from 1, 10, and 100 Hela cells. The CHAPS buffer was employed as the blank. The diluted extracts corresponding to 100 Hela cells (A) or crude lysates of 100 Hela cells (B) were preheated at 95 °C for 5 min to deactivate telomerase activity, and were used as the negative controls, respectively. (C) POI values of the real-time fluorescence curves in (A) produced by the diluted extracts equivalent to 1, 10, and 100 Hela cells. (D) Corresponding POI values of the real-time fluorescence curves in (B) produced by the crude lysates from 1, 10, and 100 Hela cells. The error bars represent standard deviation of ten replicative tests for 1 Hela cell, 5 replicative tests for 10 Hela cells, and 3 replicative tests for 100 Hela cells.

To evaluate the practicality, the proposed telomerase assay was applied to the detection of telomerase activity in different numbers of Hela cancer cells. Firstly, the crude extract prepared from 10^6^ Hela cells was serially diluted with CHAPS buffer to the amounts corresponding to 100, 10, and a single cell. The diluted extract corresponding to 100 Hela cells was preheated at 95 °C for 5 min to deactivate telomerase activity, in order to be used as the negative control. The diluted extracts and the negative control were simultaneously detected with the proposed telomerase assay, according to the procedures described in the standard protocols (ESI†). As depicted in Fig. 3A, the well-defined real-time fluorescence signals can be observed from the diluted extracts corresponding to 1–100 Hela cells, indicating that the ultrahigh sensitivity in detecting telomerase activity equivalent to a single cell can be achieved with the proposed assay. There is a good linear relationship between the POI values and the logarithm of cell numbers (*N*) with the correlation equation of POI = 48.69 – 5.41 lg *N* and the correlation coefficient *R*^2^ = 0.999. Meanwhile, both the blank and the negative control do not produce any observable fluorescence signals, so the components in the CHAPS lysis buffer (such as surfactants) and the diluted extracts (such as proteins) do not interfere with the detection of telomerase activity, indicating a high specificity of the proposed assay. It should be noted that the detection of the diluted extracts cannot reflect the cellular heterogeneity because such measurements may mask the differences in telomerase activities among individual cells. Owing to the ultrahigh sensitivity and specificity, the feasibility of the proposed SPEA strategy for the detection of cellular heterogeneity arising from cell-to-cell variations of telomerase activities was subsequently investigated. For this study, the proposed assay was directly applied to detecting telomerase activity in the crude lysates of a single Hela cell, 10 cells and 100 cells, respectively. As demonstrated in Fig. 3B, telomerase activity in concentrations as low as a single Hela cell can also be well detected and the POI values are linearly dependent on the logarithm of the cell numbers. The correlation equation is POI = 48.78 – 5.58 lg *N* and *R*^2^ = 0.993. The linear correlation coincides well with that for the detection of the diluted extracts. To quantitatively assess the detection results, the relative telomerase activity (RTA) was calculated by using the linear relationship shown in Fig. 2B as the relative standard curve. Therefore, the RTA is estimated as the corresponding copy number of SLTSR8. By using the RTAs, the relative standard deviation (RSD) for ten-time repetitive measurements of telomerase activity in the diluted extracts equivalent to a single cell and in the crude lysates of a single cell are 16.1% and 65.7%, respectively. It is worth noting that the RSDs of telomerase activity measurements from the cell lysates are much larger than that from the diluted extracts. The diluted extracts were prepared through a series of dilutions from the same pooled sample. Therefore, the resulting errors arising from the diluted extracts should come from the experimental errors, including the pipetting processes and the amplification reaction. The direct detection of the cell lysates is performed with the same experimental procedures. Therefore, the large errors in measurement of the cell lysates should reflect the cell-to-cell variation. As the experimental errors related to using this method are much smaller than the cell-to-cell variation, the proposed assay should be accurate enough for the detection of telomerase activity in a single cell and for detection of cellular heterogeneity.

### 4. Specificity of the proposed telomerase assay

To prove that the responses of the cell samples were triggered specifically by telomerase, the amplification products of SPEA were characterized by polyacrylamide gel electrophoresis (PAGE). As shown in Fig. S7 (ESI†), after incubation of SLTS with the Hela cell extracts, the amplification products showed multiple bands in ladder-like patterns, which are consistent with the prediction of SPEA products demonstrated in Fig. 1. Moreover, the ladder-like patterns of the SLTSR8 amplification products are almost the same as that of Hela cell extract-incubated SLTS, indicating that the amplification products indeed originated from the telomerase-elongated SLTS and the proposed assay is reliable for the detection of telomerase activity.

**Fig. 4:**
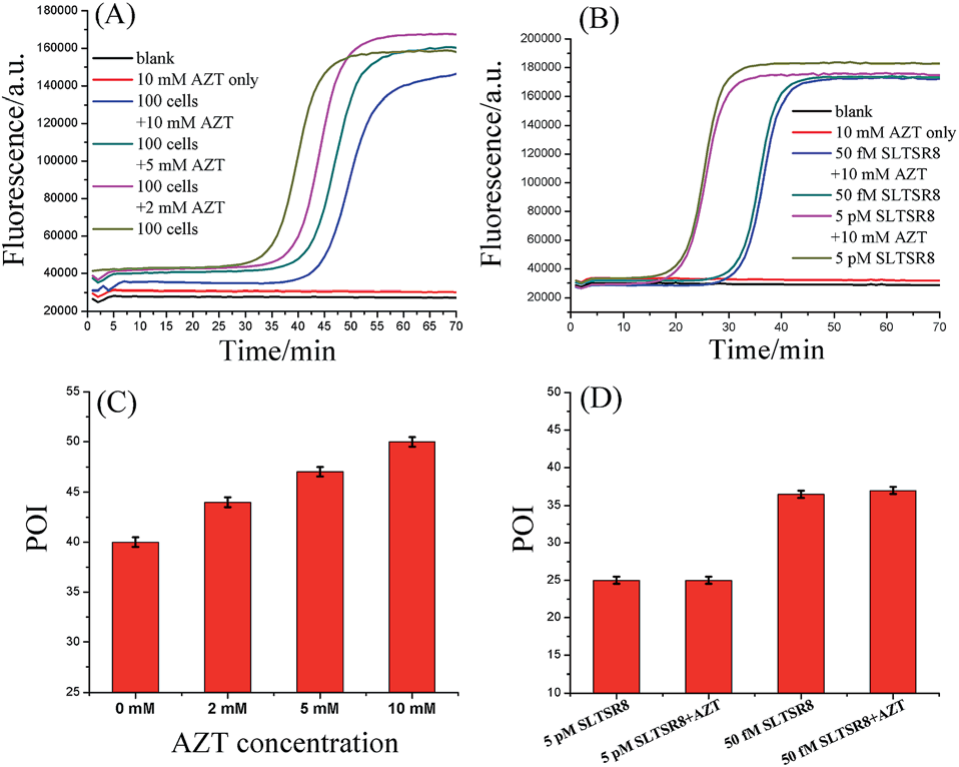
Effect of AZT on the telomerase assay. (A) Real-time fluorescence curves of diluted extract equivalent to 100 Hela cells in the presence of different concentrations of AZT. (B) Real-time fluorescence curves of 50 fM and 5 pM SLTSR8 in the presence of 10 mM AZT. (C) Corresponding POI values of the real-time fluorescence curves displayed in (A). (D) Corresponding POI values of the real-time fluorescence curves displayed in (B). The CHAPS buffer was employed as the blank. Error bars represent the standard deviation of three replicative tests.

To further test the specificity of the SPEA-based telomerase assay, different amounts of 3′-azido-3′-deoxythymidine (AZT), a known specific inhibitor of telomerase,9*e* were added into the diluted extracts equivalent to 100 Hela cancer cells. The mixtures were then detected with the SPEA-based assay. As shown in Fig. 4A, with increasing the concentration of AZT, the reaction time at which the fluorescence rises significantly above the background is obviously increased, resulting in the gradually increased POI values. The effect of AZT on the detection of synthetic SLTSR8 by the SPEA system is also investigated. As shown in Fig. 4B, the SLTSR8 and the mixture of SLTSR8 and AZT produce almost the same real-time fluorescence signals, clearly revealing that AZT specifically suppresses the telomerase activity in the extracts of Hela cells, while showing negligible effects on the SPEA system. These experimental results confirm the specificity of the SPEA-based telomerase assay and suggest its potential application for screening telomerase inhibitors as targeted anticancer drugs.

### 5. Evaluation of the generality of the SPEA-based telomerase assay

**Fig. 5:**
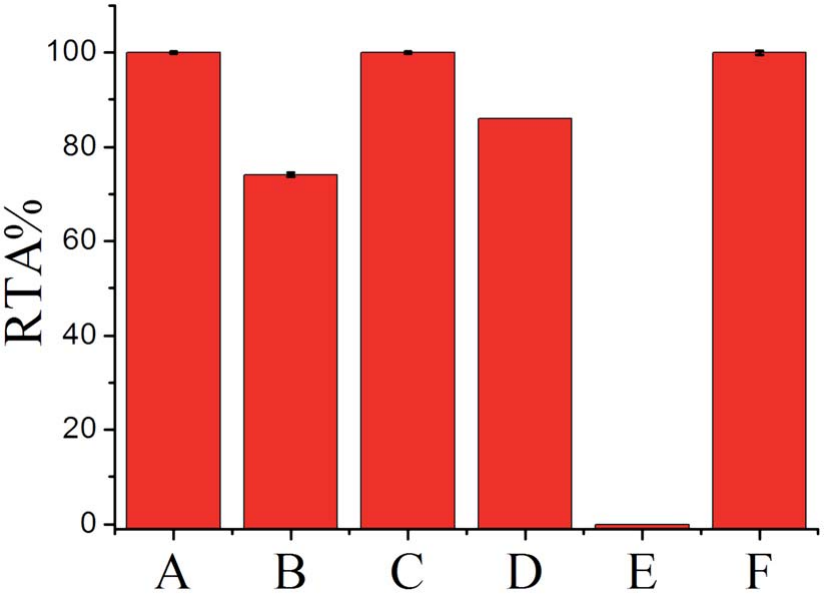
The relative telomerase activities (RTAs) of 100-cell lysates from Hela (A), CEM (B), HCT-116 (C), MCF-7 (D), MRC-5 (E), and the mixture of Hela and MRC-5 (F). The RTA of the Hela cell lysate is normalized to 100%.

To further confirm the generality and reliability of the proposed assay for telomerase detection, different cancer cell lines, including Hela cells, leukemia cells (CEM), colon cancer cells (HCT-116), breast cancer cells (MCF-7), and normal cell line (MRC-5) were simultaneously detected with the proposed assay. As demonstrated in Fig. 5, all the cancer cell lines showed positive telomerase activities, whereas the normal cell line did not generate any fluorescence signal. Additionally, after mixing with the same amount of MRC-5 normal cells, the detection result of telomerase activity from Hela cells was almost the same as that from the same amount of Hela cells alone, further demonstrating the robustness of the proposed assay. Moreover, we further applied the proposed SPEA strategy to the detection of telomerase activities in diluted extracts equivalent to a single cell and in the crude lysates of a single cell for the different cell lines (Fig. S8 in the ESI†). Similar to the results shown in Fig. 3 for Hela cells, for the other tested cancer cell lines (CEM, MCF-7 and HCT-116), the RSDs for RTA measurements from the single cell lysates were all much larger than those from the diluted cell extracts. These results clearly indicate that the SPEA strategy is well suited for the detection of the cell-to-cell variation of telomerase activities in different cell lines at the single-cell level.

## Conclusions

By strategically designing SLTS and SLP, we have developed a novel SPEA system to detect the telomerase-elongated telomeric repeat DNAs, which can be performed under isothermal conditions with a one-step reaction and using one-type of DNA polymerase. Taking advantage of the ultrahigh sensitivity and near-zero nonspecific amplification, the SPEA-based telomerase assay can realize the accurate detection of telomerase activity from individual cells. As an almost universal marker for human cancers,15*a* telomerase is a promising biomarker for cancer diagnostics and therapeutics. Therefore the ultrasensitive, simple, and reliable telomerase assay shows great potential for use in clinical tests and for telomerase-related fundamental research. On the other hand, the isothermal exponential amplification is a powerful tool, not only for telomerase detection but also for detection of various genetic biomarkers. The nonspecific amplification is always a key problem that limits the sensitivity and specificity of detection of these biomarkers. Owing to the ultrahigh specificity, we believe the new SPEA system will also benefit assays for the detection of genetic biomarkers, and will find wide applications in biomedical research and molecular diagnostics.

## Supplementary Material

Supplementary information
