# Supplementary material for: Ultrasensitive detection of telomerase activity in a single cell using stem-loop primer-mediated exponential amplification (SPEA) with near zero nonspecific signal
Source: Chem Sci. 2016 Apr 28;7(8):4945–50. doi: 10.1039/c6sc00802j (PMC6018513; doi:10.1039/c6sc00802j)
Supplement: Supplementary file 1 [file SC-007-C6SC00802J-s001.pdf]

Electronic Supplementary Material (ESI) for Chemical Science.
This journal is © The Royal Society of Chemistry 2016

Electronic Supplementary Information

Ultrasensitive Detection of Telomerase Activity in a Single Cell Using Stem-loop Primer-mediated Exponential Amplification (SPEA) with Near Zero Nonspecific Signal

Honghong Wang, Hui Wang, Chenghui Liu,* Xinrui Duan and Zhengping Li*

Key Laboratory of Applied Surface and Colloid Chemistry, Ministry of Education; Key Laboratory of Analytical Chemistry for Life Science of Shaanxi Province; School of Chemical and Chemical Engineering, Shaanxi Normal University, Xi'an 710062, Shaanxi Province, P. R. China.

Email: liuch@snnu.edu.cn; lzpbd@snnu.edu.cn

Content List

1. Materials and apparatus
2. Cell culture
3. Preparation of telomerase extracts
4. Preparation of cell lysates
5. Standard protocols of telomerase assay
6. Optimization of the amount of Bst DNA polymerase
7. Optimization of the amount of stem-loop primer (SLP)
8. Optimization of the temperature of SPEA reaction
9. Evaluation and explanation of the near-zero nonspecific amplification
10. Characterization of SPEA products with gel electrophoresis
11. Detection of telomerase activities in the diluted cell extracts and crude cell lysates from CEM, HCT-116, MCF-7 and MRC-5 cell lines

1. Materials and apparatus

The CHAPS lysis buffer was purchased from Millipore Co., Ltd (USA). Bst DNA polymerase large fragment (8 U/μL) and 10× ThermoPol reaction buffer (200 mM Tris-HCl, 100 mM KCl, 100 mM $(NH_4)_2SO_4$, 20 mM $MgSO_4$ and 1% Triton X-100, pH 8.8 @25°C) were purchased from New England Biolabs (USA). SYBR Green I (20 ng/μL stock solution in DMSO) was purchased from Xiamen Bio-Vision Biotechnology (Xiamen, China). The betaine was obtained from Sigma (USA). 3′-azido-3′-deoxythymidine (AZT) was purchased from TCI Co. Ltd (Shanghai, China). Magnesium chloride ($MgCl_2$), Tween-20, potassium chloride (KCl), ethylene glycol tetraacetic acid (EGTA), Trihydroxymethyl aminomethane (Tris) and 4S Red Plus nucleic acid stain were obtained from Sangon Biotech Co. Ltd. (Shanghai, China). The SLTSR8 oligonucleotide was synthesized by Integrated DNA Technologies. The other oligonucleotides used in this work, RNase inhibitor, dNTPs, RNase-free water, 20 bp DNA ladder and 6× nucleic acid sample loading buffer were purchased from TaKaRa Biotechnology Co. Ltd. (Dalian, China). All the reagents were of analytical grade and were used as received without further purification. All the oligonucleotides were purified by PAGE. The sequences of the oligonucleotides were given in the **Table S1**. The Hela, MCF-7, CEM, HCT-116 and MRC-5 cell lines were purchased from the cell bank of Chinese Academy of Sciences (Shanghai, China). The real-time fluorescence measurements were performed with the StepOne Real-Time PCR system (Applied Biosystems, USA).

Table S1. The sequences of the oligonucleotides used in this work.

Name	Sequences (5′-3′ direction)
SLTSR8	CGACAGCAGAGGATTTGTTGTGTGGAAGTGTGAGCGGATTTTCCTCTGCTG<br>F1c F2 F1<br>TCGTTTTAATCCGTCGAGCAGAGTT(TTAGGG)$_8$<br>TS
SLTS	CGACAGCAGAGGATTTGTTGTGTGGAAGTGTGAGCGGATTTTCCTCTGCTG<br>F1c F2 F1<br>TCGTTTTAATCCGTCGAGCAGAGTT<br>TS
SLP	ATCGTCGTGACTGTTTGTAATAGGACAGAGCCCCGCACTTCAGTCACGACG<br>B1c B2 B1<br>ATTTTCCCTAACCCTAACCCTAACCC
FIP	CGACAGCAGAGGATTTGTTGTGTGGAAGTGTGAGCGGA<br>F1c F2
BIP	ATCGTCGTGACTGTTTGTAATAGGACAGAGCCCCGCAC<br>B1c B2

2. Cell culture

The Hela, MCF-7, HCT-116 and MRC-5 cell lines were cultured in 5 mL DMEM Medium (GBICO, Cat. 12100-046) containing 10% (v/v) fetal calf serum (GBICO, Cat. 1600036), 1% $NaHCO_3$, 100 U/mL penicillin, 100 μg/mL streptomycin and 3 mmol/L L-glutamine. The CEM cell line was cultured in 5 mL RPMI 1640 Medium (Sigma, Cat. R6504) containing 10% (v/v) fetal bovine serum, 1% $NaHCO_3$, 100 U/ml penicillin, 100 μg/ml streptomycin and 3 mmol/L L-glutamine. All of the cell lines were maintained at 37 °C under a humidified atmosphere containing 5% CO_2.

3. Preparation of telomerase extracts

The cultured Hela cells or other cells were centrifuged at 700 rpm for 5 minutes to remove the culture medium after trypsinization (0.2% trypsin, 1 mM EDTA, Invitrogen), followed by washing three times with the cold D-PBS buffer (10 mM sodium phosphate buffer, 0.1 M NaCl, pH 7.4 @25°C). 10^6 adherent Hela cells or other cells were collected respectively into a centrifuge tube and then the cells were pelleted by centrifuged at 2000 rpm for 10 min. Each cell pellet (containing 10^6 cells) was suspended in the 200 μL cold CHAPS lysis buffer (5000 cells/μL), incubated on ice for 30 min and then centrifuged for 30 min at 12000 rpm at 4°C. The telomerase extracts in the supernatant were frozen and stored at -80°C as the stock extracts. The samples for detection of telomerase activity were prepared by appropriate dilution of the stock extracts with CHAPS buffer.

4. Preparation of cell lysates

The cultured Hela cells or other cells were centrifuged at 700 rpm for 5 minutes to remove the culture medium after trypsinization (0.2% trypsin, 1 mM EDTA, Invitrogen), washed three times with cold D-PBS buffer, and then re-suspended in the cold D-PBS buffer at a concentration of 10^4 cells per μL. For the telomerase activity analysis of 100 cells, the cell suspension was serially diluted to 100 cells per μL in the cold D-PBS buffer. 1 μL diluted cell suspension containing 100 cells was pipetted to 1 μL cold CHAPS lysis buffer, and then incubated for 30 min on ice. For the telomerase activity analysis of 10 cells or 1 cell, firstly, the cell suspension was serially diluted to 10 cells per μL or 1 cell per μL in the cold D-PBS buffer, 1 μL diluted cell suspension (containing 10 cells or 1 cell) was transferred to the microslide, and then 10 cells or 1 cell was counted and manipulated by using Narishige micromanipulator system equipped on an Olympus IX53 inverted microscope with a monitor. Subsequently the 10 cells or 1 cell were dropped into 1 μL cold CHAPS lysis buffer, incubated for 30 min on ice. The cell lysates were then immediately used as the samples to detect the telomerase activity.

5. Standard protocols of telomerase assay

The cell lysates or 1 μL diluted extracts were added to the reaction mixture containing 0.2 μM stem-loop telomerase substrate (SLTS) primer, 0.5 mM dNTPs, 1.6 U/μL RNase inhibitor and telomerase reaction buffer (20 mM Tris-HCl, 1.5 mM $MgCl_2$, 63 mM KCl, 1 mM EGTA, 0.05% Tween-20) with the final volume of 5.0 μL. Then the mixture was incubated for 30 min at 37 °C to elongate the SLTS primer, and finally heated to 95 °C for 5 min to inactivate telomerase activity. For the detection of SLTSR8 model target, SLTS was replaced by SLTSR8 of different concentrations without the cell lysates or the diluted extracts. The CHAPS buffer was used as the blank, which was detected with the same procedures in the absence of cell lysates, diluted extracts or SLTSR8.

After the telomerase reaction, the reaction products were mixed with a standard SPEA reaction solution containing 200 mM betaine, 1× ThermoPol reaction buffer (20 mM Tris-HCl, 10 mM KCl, 10 mM $(NH_4)_2SO_4$, 2 mM $MgSO_4$, 0.1% Triton X-100), 250 μM dNTP, 0.8 μM FIP, 0.8 μM BIP, 0.1 μM stem-loop primer (SLP), 0.4 ng/μL SYBR Green I and 0.4 U/μL Bst DNA polymerase with a final volume of 10.0 μL. Then, the final mixture solution was immediately put into the StepOne Real-Time PCR system to perform the SPEA reaction at 65 °C. The real-time fluorescence intensity was simultaneously monitored at intervals of 1 min.

6. Optimization of the amount of Bst DNA polymerase

The SPEA reaction relied on auto-cycling strand displacement DNA synthesis that was catalyzed by Bst DNA polymerase with displacement activity. So the amount of Bst DNA polymerase was an important parameter for the SPEA-based telomerase assay. As described in the paper, a synthetic telomerase-elongated product SLTSR8, corresponding to SLTS elongated with eight telomeric repeats (TTAGGG), was employed as a model to optimize the experiment conditions. Therefore, the effect of amount of Bst DNA polymerase was investigated by detection of SLTS8 at different concentrations with the SPEA-based assay. As shown in Fig. S1 (A-D), with increasing the amount of Bst DNA polymerase from 0.16 ~ 0.64 U/μL, the SPEA reaction was correspondingly accelerated, resulting in the decrease of POI values of SLTSR8. However, the difference of the POI values between 10 aM, 100 aM and 1 fM SLTSR8 gradually decreased when Bst DNA polymerase was increased from 0.4 to 0.64 U/μL. When 0.16 U/μL Bst DNA polymerase was employed, the SPEA reaction needed long time greater than 90 min to produce detectable signals. Taking into consideration of both reaction time and detection sensitivity, 0.4 U/μL was selected as the optimum amount of Bst DNA polymerase for the SPEA-based telomerase assay.

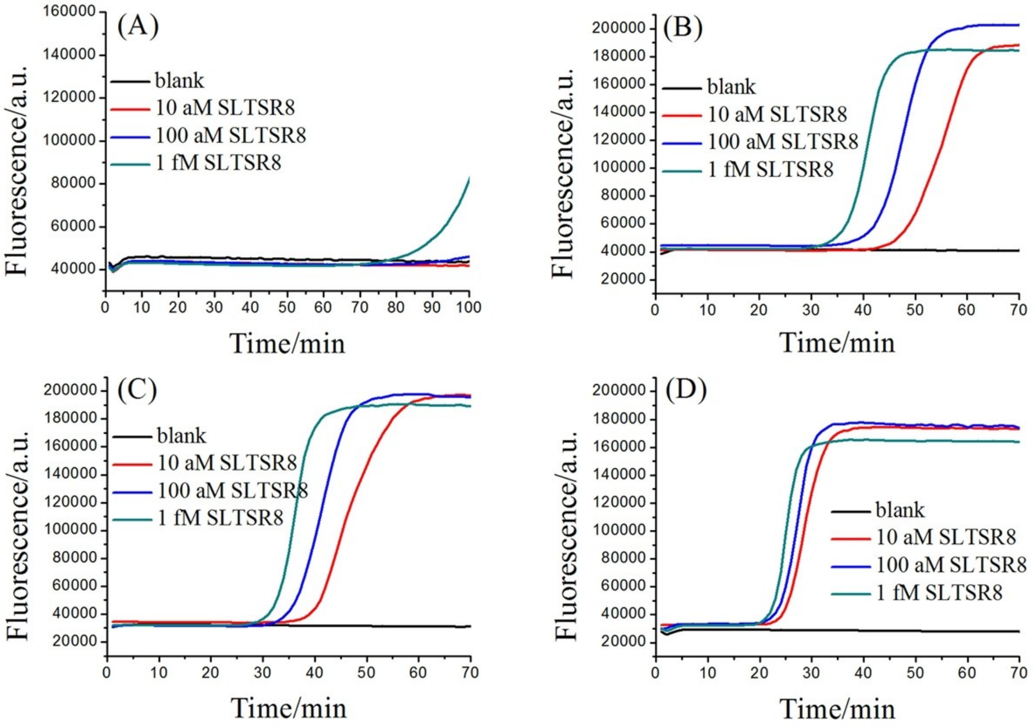

Fig. S1. Effect of the amount of Bst DNA polymerase on the detection of SLTSR8. The blank was detected in the same procedure in the absence of SLTSR8. The SPEA reaction and real-time measurement of fluorescence signals were carried out according to the procedures described in the standard protocols of telomerase assay except the amount of DNA polymerase, which was (A) 0.16 U/μL, (B) 0.4 U/μL, (C) 0.52 U/μL, and (D) 0.64U/μL, respectively.

7. Optimization of the amount of stem-loop primer (SLP)

To investigate the influence of the amount of SLP, 10 aM, 100 aM and 1 fM SLTSR8 were respectively detected with the SPEA-based assay by using SLP of different concentrations. As depicted in Fig. S2 (A-C), with increasing the SLP concentration from 50 nM to 200 nM, the SPEA reaction was gradually speeded up. However, the POI values of SLTSR8 decreased very little. The differences of the POI values between 10 aM, 100 aM, and 1 fM SLTSR8 reached their maximum when 100 nM SLP was used. Therefore, 100 nM SLP was employed for the SPEA-based assay.

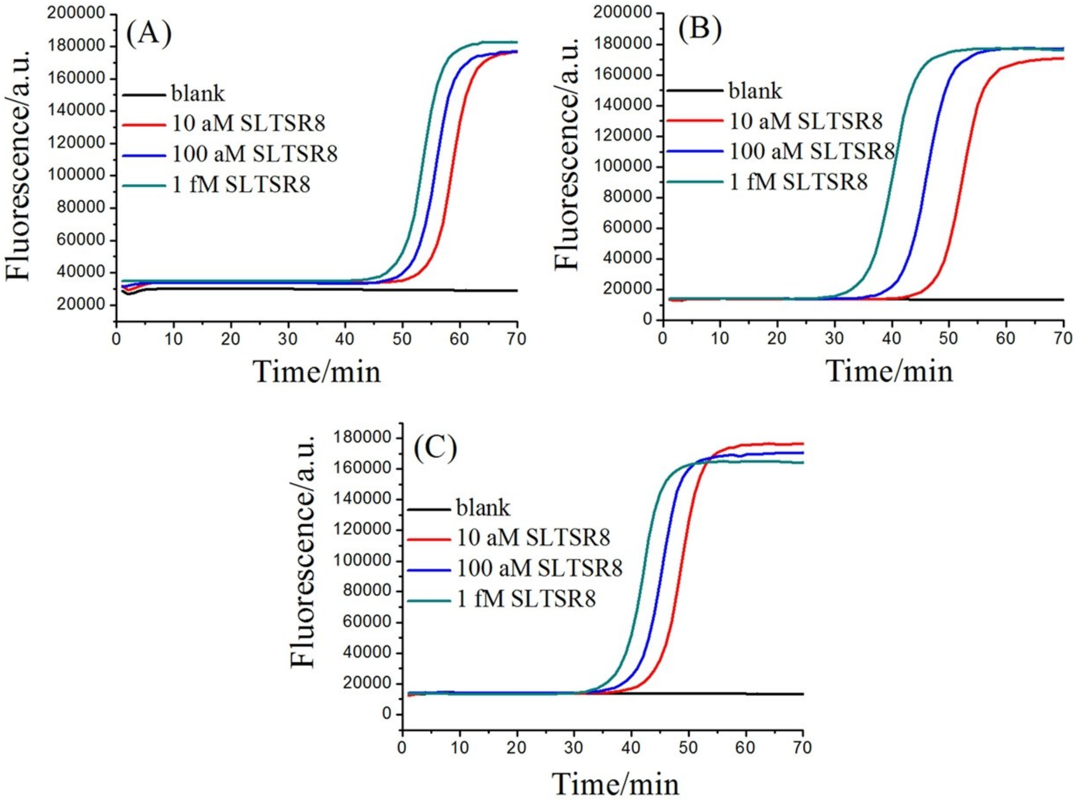

Fig. S2. Influence of the amount of the SLP. The real-time fluorescence curves were produced by 1 fM, 100 aM, 10 aM SLTSR8 and the blank with SPEA reaction. The SPEA reaction and measurement of fluorescence were carried out according to the procedure described in the standard protocols of telomerase assay except the amount of the SLP, which was (A) 50 nM, (B) 100 nM, (C) 200 nM, respectively.

8. Optimization of the temperature of SPEA reaction

The temperature of SPEA reaction was closely associated with the reaction speed and the sensitivity for detection of telomerase activity. The influence of temperature of SPEA reaction was investigated by simultaneously detecting the blank, 10 aM, 100 aM and 1 fM SLTSR8 with the SPEA-based assay at different reaction temperatures. As demonstrated in Fig. S3(A-B), when the reaction temperature was elevated from 60 °C to 65 °C, the POI values of SLTSR8 were shortened about 15 min and the difference of the POI values between 10 aM, 100 aM and 1 fM SLTSR8 were increased. However, when the SPEA was performed at 70 °C, as shown in Fig. S3C, no detectable fluorescence signals could be observed, indicating that no SPEA reaction took place at 70 °C. It should be reasonable that the melting temperature (Tm) of the double stranded stem in SLP and SLTSR8 is less than 70 °C. So the stem-loop structure of SLP and SLTSR8 will be destroyed. Therefore, 65 °C was selected as the optimized temperature of SPEA reaction in this work.

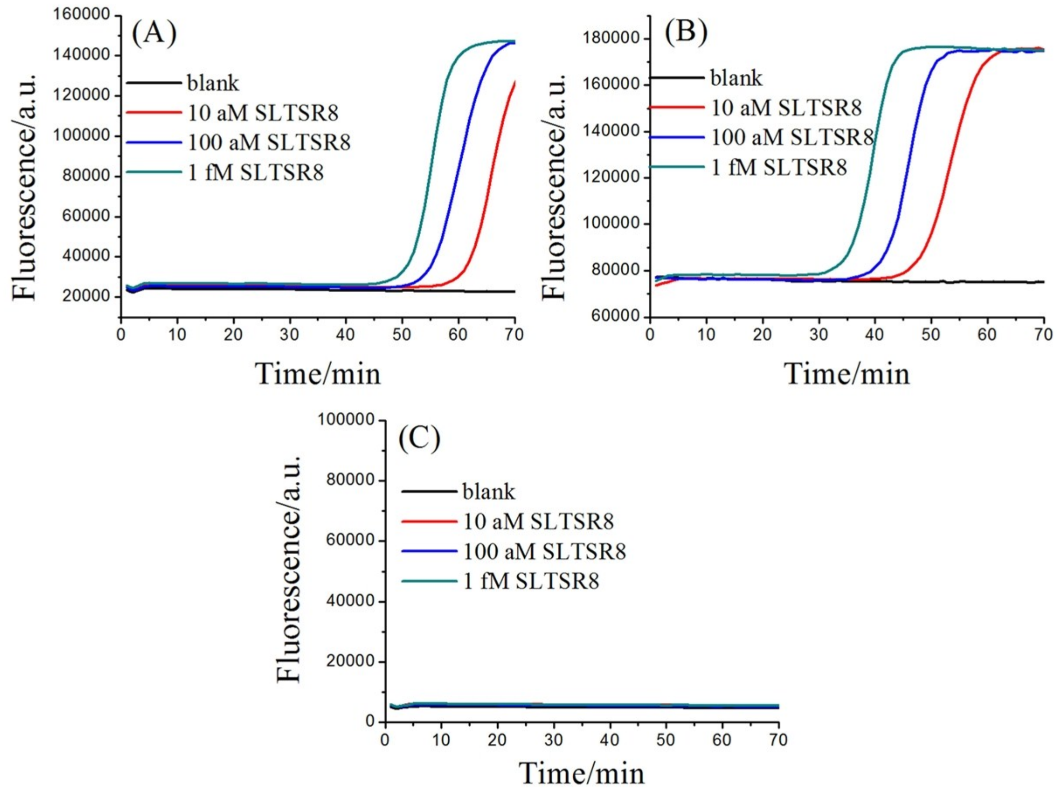

Fig. S3. Influence of the SPEA reaction temperature. The real-time fluorescence curves were respectively produced by 1 fM, 100 aM, 10 aM SLTSR8 and the blank with the SPEA reaction. The reaction temperature was (A) 60 °C, (B) 65 °C, and (C) 70 °C, respectively. Other experiment conditions were the same as described in the standard protocols of telomerase assay.

9. Evaluation and explanation of the near-zero nonspecific amplification

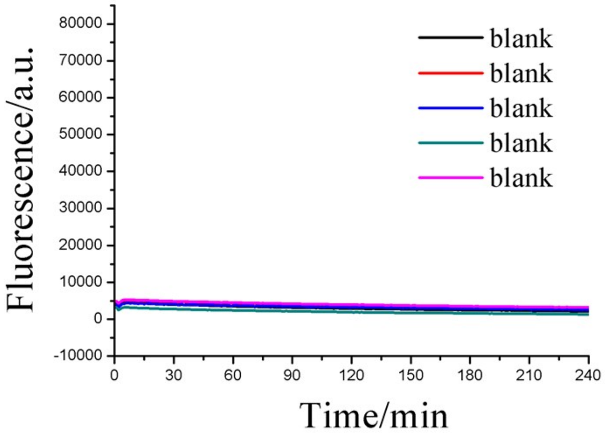

Fig. S4. Real-time fluorescence curves of five replicative measurements of blank control in the SPEA reaction.

As shown in Fig. S4, the blank fluorescence signal of the SPEA reaction still maintains a constant straight line even when the reaction time extends up to 240 min, indicating the near zero nonspecific amplification in the proposed SPEA strategy.

The efficient preclusion of non-specific amplification in this work is mainly due to the ingenious probe design in the SPEA system. Firstly, in the proposed SPEA system, the double stem-loop DNAs are the essential starting materials for the subsequent loop-mediated isothermal signal amplification. As illustrated in Fig. 1, if telomerase is absent, the SLTS cannot be elongated to produce the telomere repeat, and thus the telomere repeat-templated extension of SLP cannot occur at all. Consequently, no double stem-loop DNAs can be formed to initiate the subsequent signal amplification reaction. Secondly, in the proposed SPEA system, the sequences of FIP and BIP are also rationally designed to avoid nonspecific signal amplification. FIP has the same sequence with F1c and F2 of SLTS, and BIP has the same sequence with B1c and B2 of SLP. As such, when telomerase is absent, the four co-existed nucleic acid sequences (SLTS, SLP, FIP, BIP) in the SPEA system will not hybridize with each other to produce any nonspecific amplification reaction due to their stringent sequence design.

On the other hand, for the conventional thermal cycle-based PCR amplification, the formation of primer-dimer (self-dimer or hetero-dimer) of the PCR primers during the thermal cycles is the main source of nonspecific amplification. The primer-dimers can form in the annealing step of PCR when some bases in the primers are complementary. As illustrated in Fig. S5, even the formation of very few primer-dimers may ultimately generate significant false positive results after exponential amplification with the PCR thermal cycles. In contrast, in the SPEA system, the exponential amplification is performed under isothermal conditions. As illustrated in Fig. S6, even if the primers of the SPEA could also form the self-dimer or hetero-dimer, the self-dimer or hetero-dimer of SPEA primers can only extend one time and then the primer extension will be stopped under the constant temperature, which cannot initiate the subsequent signal amplification and cannot generate obvious nonspecific signals. Therefore, from above we can concluded that the near zero nonspecific signal in this study is reasonable due to the rational probe design and the isothermal nature of the exponential amplification in SPEA.

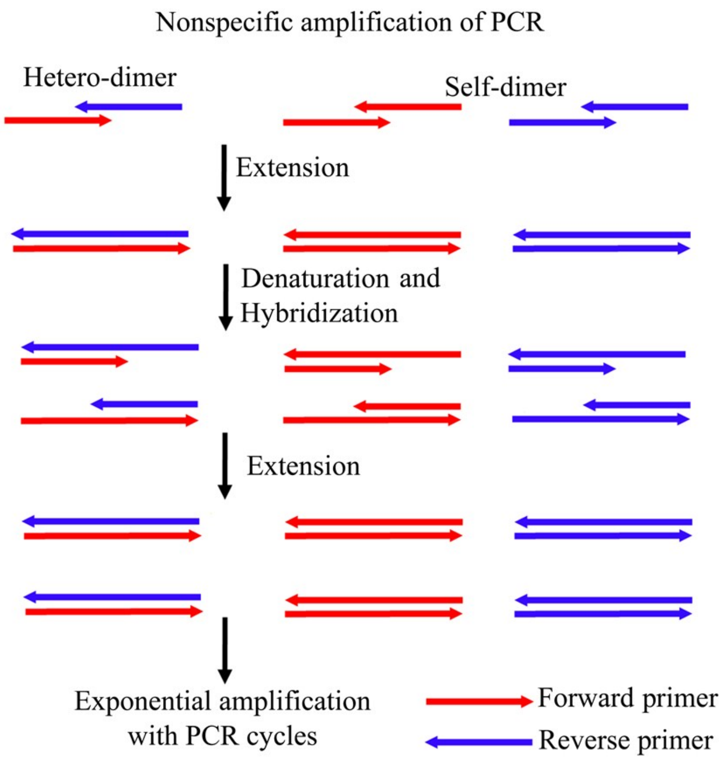

Fig. S5. Schematic illustration of the nonspecific amplification by the formation of self-dimer or hetero-dimer of PCR primers during the PCR thermal cycles.

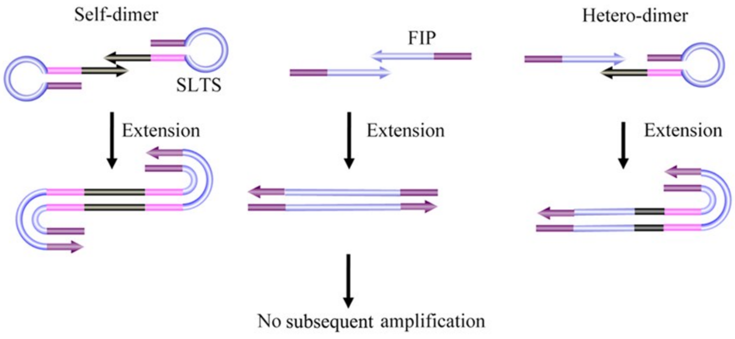

Fig. S6. The potential effect of the primer-dimer formation on the nonspecific amplification of the SPEA system.

10. Charaterization of SPEA products with gel electrophoresis

To further support the proposed SPEA-based telomerase assay, the amplification products of SPEA reaction were characterized by polyacrylamide gel electrophoresis (PAGE). As shown in Fig. S7, the SLTS shows a defined band corresponding to 52 bp (lane 2). After incubation of SLTS with the Hela cell extraction, as depicted in lane 3, the amplification products of SPEA reaction showed

multiple bands in ladder-like patterns. According to the principle of SPEA discussed in Fig. 1, the SPEA products are a large amount of the mixture of stem-loop DNAs with various stem lengths. So the ladder-like patterns of PAGE were well consistent with the prediction of SPEA products. Meanwhile, the negative control (lane 4) and the blank (lane 5), in which the Hela cell extract is heat-inactivated or absent, did not produce any observable bands except for the SLTS band. On the other hand, the synthetic SLTSR8 shows a band at the position corresponding to about 160 bp of dsDNA marker (lane 6). When the SLTSR8 was amplified by SPEA reaction, as shown in lane 7, the multiple bands with the ladder-like pattern could be observed from the amplification products. More importantly, the ladder-like pattern of the SLTSR8 amplification products was almost the same as that of Hela cell extract-incubated SLTS amplification products, indicating that the amplification products shown in lane 3 were indeed originated from the telomerase-elongated SLTS and the proposed assay was reliable for detection of telomerase activity.

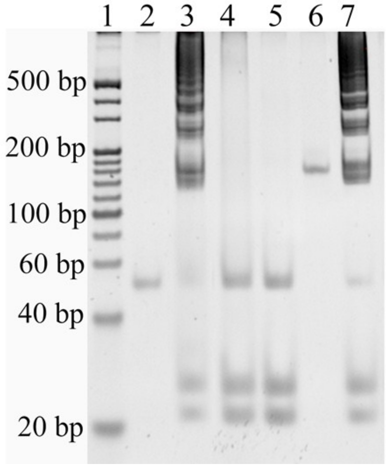

Fig. S7. Non-denaturing PAGE analysis of the amplification products of SPEA reaction. Lane 1, double strand (ds) DNA markers (20 bp DNA ladder); lane 2, SLTS (100 nM); lane 3, amplification products in the presence of cell extract; lane 4, amplification products with heat-inactivated cell extract (the negative control); lane 5, amplification products of the blank in the absence of cell extract and SLTSR8; lane 6, SLTSR8 (100 nM); lane 7, amplification products of SLTSR8 (10 pM). The gel was stained by 4S Red Plus Nucleic Acid Stain and visualized on a Gel Doc EZ Imager (Bio-Rad, USA).

11. Detection of telomerase activities in the diluted cell extracts and crude cell lysates from CEM, HCT-116, MCF-7 and MRC-5 cell lines

To test whether the proposed assay is generally applicable to the detection of cellular heterogeneity arising from cell-to-cell variations in different cell lines, the SPEA strategy was further applied to the detection of telomerase activities in different number of cancer cells (CEM,

HCT-116 and MCF-7 cell lines) as well as normal cell line MRC-5. For each tested cell line, diluted extracts equivalent to 1, 10, 100 cells and cell lysates from 1, 10, 100 cells are repectively tested according to procedures described in the standard assay protocols. The CHAPS buffer was employed as the blank while the diluted extract corresponding to 100 cells or cell lysates of 100 cells was preheated at 95 °C for 5 min to inactivate telomerase activity to be used as the negative control. As depicted in Fig. S8, the blank and the negative control do not produce any observable fluorescence signals. Similarly, the diluted extracts or lysates from 1~100 normal cell line MRC-5 also do not generate any fluorescence response. In contrast, for the tested cancer cell lines such as CEM, HCT-116 and MCF-7, the well-defined real-time fluorescence signals can be observed both from the diluted extracts and cell lysates correspongding to 1~100 cells. Notably, one can see from Fig. S8 that for these tested cancer cell lines, the variations of fluorescence responses produced by the single cell lysates are much larger than those produced by the diluted cell extracts, which is in well accordance with the results of Fig. 3 for telomerase analysis in Hela cell. By using the RTA values for quantitative evaluation of telomerase activities, the relative standard deviations (RSDs) of CEM, HCT-116 and MCF-7 for ten-time repetitive measurements of telomerase activity in the diluted extracts equivalent to a single cell are 20.4%, 16.5% and 18.4%, respectively. Meanwhile, the RSDs of CEM, HCT-116 and MCF-7 for ten-time repetitive measurements of telomerase activity in the crude lysates of a single cell are 72.5%, 69.1% and 71.2%, respectively. The RSDs of telomerase activity measurements from the single cell lysates are all much larger than those from the diluted extracts. These results suggest that the SPEA strategy is well suitable for the detection of cell-to-cell variation of telomerase activities in different cell lines at the single-cell level.

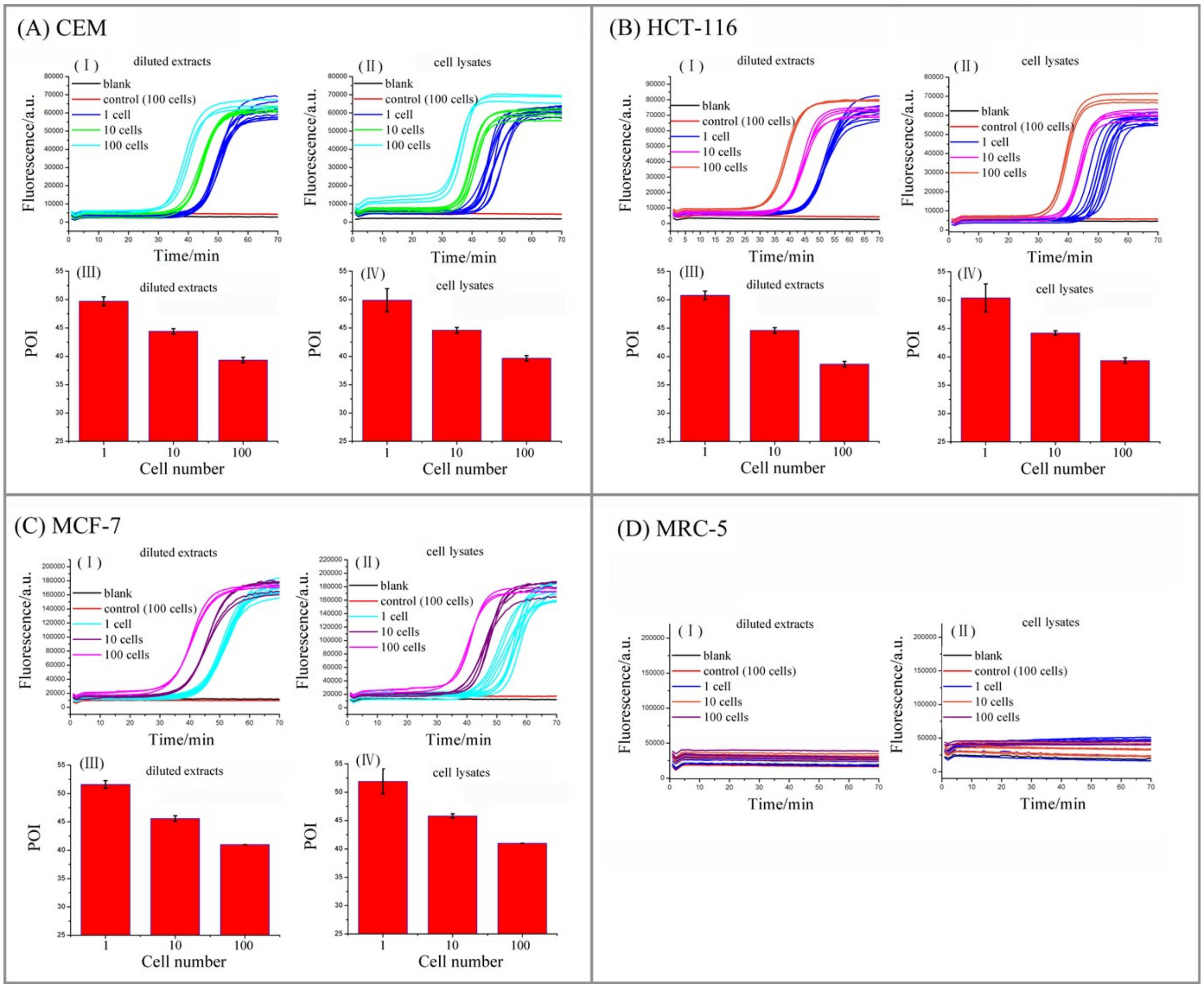

Fig. S8. Detection of telomerase activities from the diluted extracts and cell lysates of CEM (A), HCT-116 (B), MCF-7 (C) and MRC-5 (D) by the SPEA strategy. In each figure panel of A~D, image (I) displays the real-time fluorescence curves produced by diluted extracts equivalent to 1, 10, and 100 cells, while image (III) are the correspinding POI values of the fluorescence curves in image (I). In contrast, image (II) in each panel exhibits the real-time fluorescence curves for detecting the lysates from 1, 10, and 100 cells, and image (IV) shows the POI values corresponding to the fluorescence curves in image (II). Error bars indicate standard deviation of ten replicative tests for 1 cell, 5 replicative tests for 10 cells, and 3 replicative tests for 100 cells, respectively. The CHAPS buffer was employed as the blank and the diluted extract corresponding to 100 cells (in image (I)) or crude lysates of 100 cells (in image (II)) is preheated at 95 °C for 5 min to inactivate telomerase activity to be used as the negative control.
